# Up and over staged endoconduit technique for endovascular aortic aneurysm repair

**DOI:** 10.1016/j.jvscit.2024.101449

**Published:** 2024-02-13

**Authors:** Andres V. Figueroa, Mira T. Tanenbaum, Jose Eduardo Costa-Filho, Marilisa S. Gonzalez, Mirza S. Baig, Carlos H. Timaran

**Affiliations:** Division of Vascular and Endovascular Surgery, Department of Surgery, University of Texas Southwestern Medical Center, Dallas, TX

**Keywords:** Common femoral artery, Common iliac artery, Endoconduit, External iliac artery, Fenestrated/branched endovascular aneurysm repair, Iliofemoral, Peripheral arterial disease

## Abstract

Adverse iliofemoral anatomy can preclude complex endovascular aortic aneurysm repair. This study aims to describe the “up-and-over” staged endoconduit technique to improve access and avoid vascular injury before complex endovascular aneurysm repair. A staged procedure for complex endovascular aortic aneurysm repair is performed using an endoconduit (W.L. Gore & Associates). After obtaining contralateral femoral access, the extension of iliofemoral disease is assessed using angiography. The endoconduit is advanced “up and over” the aortic bifurcation and delivered percutaneously into the common femoral artery to treat a diseased access site and maintain intact the ipsilateral femoral access for future stent graft deployment. Internal iliac artery patency is maintained when feasible. During complex aneurysm repair, the endoconduit is accessed directly under ultrasound guidance using sequential dilation to avoid vascular injury. PerClose sutures (Abbott Vascular) are used to close the endoconduit femoral access site. This study found that staged “up and over” endoconduit creation is a useful technique before complex endovascular aneurysm repair in patients with adverse iliofemoral anatomy. Avoiding accessing the main femoral access site during the first stage prevents vascular or access site injuries and allows for both iliac and femoral disease to be addressed.

Endovascular repair has emerged as the first-line treatment option for patients with aortic aneurysms with significant short-term advantages compared with open repair.^1^, ^2^, ^3^, ^4^, ^5^, ^6^ Complex endovascular aortic aneurysm repair (EVAR) and thoracic endovascular aortic aneurysm repair (TEVAR) often require a large femoral access to advance the main device. The presence of narrow or diseased iliac and femoral arteries and tortuous access can preclude endovascular repair and increase the risk of access-related complications.^7^^,^^8^ Previous data suggest adverse iliofemoral anatomy is related to an increased risk of iliac rupture, failure to advance the device, and increased late mortality.^8^^,^^9^ Some strategies that involve open and endovascular conduits have been described previously with favorable success. Nevertheless, periprocedural complications, including bleeding, wound complications, delayed endovascular repair, and prolonged hospitalization, are not negligible.^6^^,^^10^, ^11^, ^12^ In our practice, staged endoconduits (ECs) are extended through the common femoral artery (CFA) to improve access and avoid vascular injury at the future access site. We describe the “up-and-over” staged EC technique before complex EVAR.

## EC Technique

**Fig 1:**
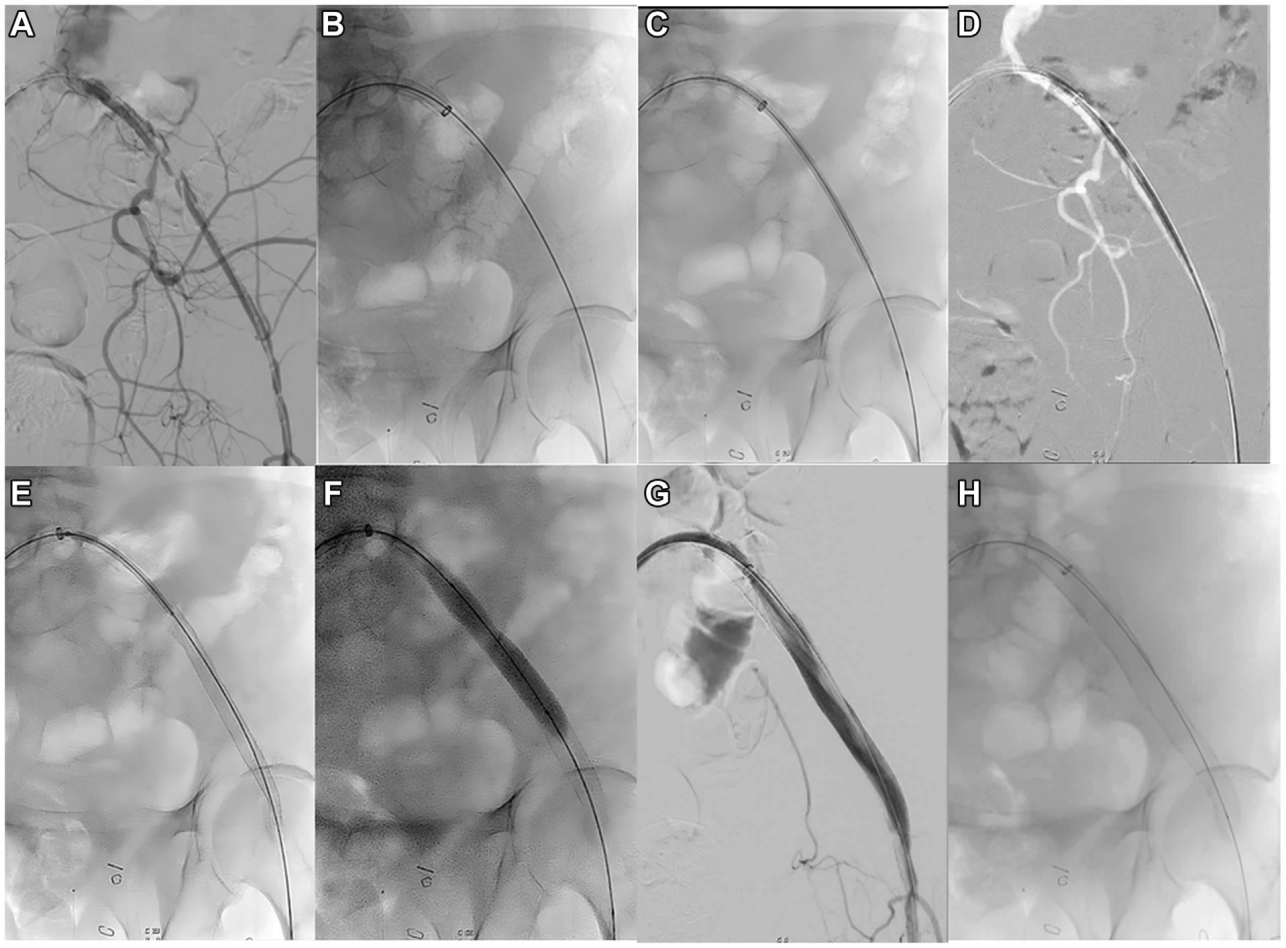
**A,** After obtaining contralateral femoral access, an angiogram is performed to evaluate the iliofemoral anatomy in the ipsilateral femoral access. **B,** The access is then upsized with an 8F or 12F Ansel sheath. **C,** A self-expanding covered stent is then advanced up and over the aortic bifurcation. **D,** Adequate positioning of the endoconduit (EC) is confirmed under fluoroscopy visualization. **E,** The EC is then deployed into the common femoral artery (CFA). **F,** After stenting, high-pressure balloon angioplasty of the stented vessel is performed using the “pave and crack” technique. **G,** Patency is confirmed using iliofemoral angiography. **H,** EC coverage of the future access site is evident under fluoroscopy visualization.

**Fig 2:**
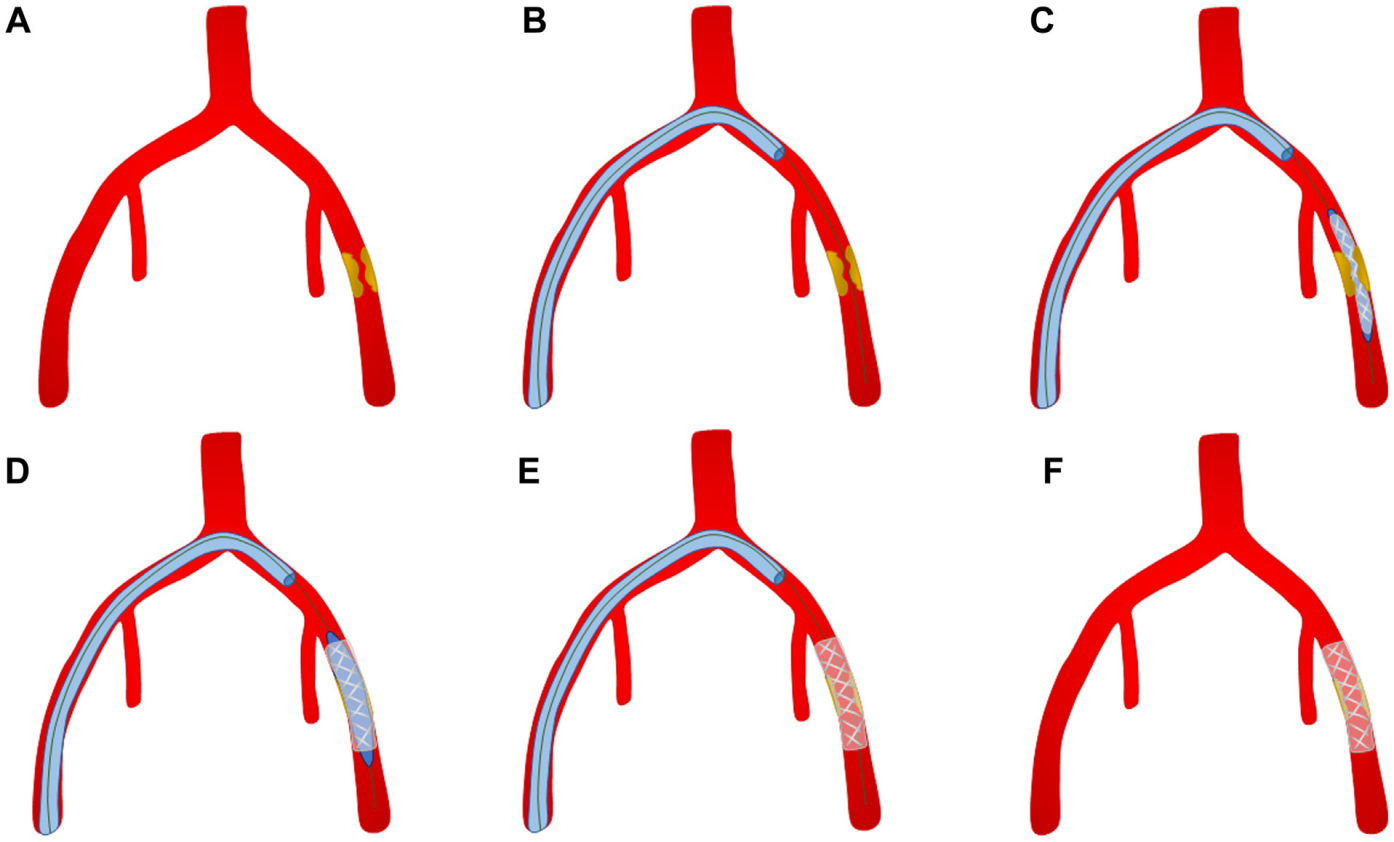
**A,** Common femoral artery (CFA) disease on the left side. **B,** An RBI catheter or similar and a Glidewire are advanced up and over the bifurcation into the CFA. **C,** The self-expanding covered stent is then advanced and deployed into the CFA. **D,** High-pressure balloon angioplasty of the stented vessel is performed using the “pave and crack” technique. **E,** Patency is confirmed using angiography. **F,** Self-expanding covered stent deployed into the CFA to be accessed during the complex EVAR or TEVAR procedure.

Contralateral femoral access is obtained under ultrasound guidance using a 5F sheath. Access is upsized to an 8F sheath, followed by the placement of two preclosing sutures, if necessary. An RBI catheter (Merit Medical) or a similar curved catheter and a Glidewire (Terumo Interventional Systems) are advanced up and over the aortic bifurcation into the contralateral common iliac artery (CIA). Adverse anatomy is assessed with pelvic angiography (Fig 1, *A*). After upsizing to an 8F or 12F Ansel sheath (Cook Medical; Fig 1, *B*), an EC (W.L. Gore & Associates) is advanced over a stiffer support wire and deployed into the CFA (Fig 1, *C-E*). If areas of severe stenosis are present, predilatation with a smaller balloon could be required. Although extension into the CFA is not always required if disease is limited to the iliac arteries, in most cases, EC extension into an area proximal to the CFA bifurcation will be required. Internal iliac artery (IIA) patency is maintained when feasible. High-pressure balloon angioplasty of the stented vessel is performed using the “pave and crack” technique (Fig 1, *F*). Patency is confirmed using angiography (Fig 1, *G* and *H*). Residual stenosis at the origin of the IIA could require stenting of the area with a bare self-expanding stent. Depending on the access required, an 8-mm, 9-mm, or 10-mm self-expanding stent is used. The 8-mm Viabahn stent graft is usually reserved for lower profile devices (ie, ≤18F). Larger Viabahn stent grafts (9-10 mm) are used for larger profile devices. For a 24F device, we normally use a 10-mm Viabahn EC. Oversizing of the ECs is preferable to avoid disruption of the stent graft or the iliac and femoral vessels during the second stage procedure (complex EVAR or TEVAR). Finally, access is closed using the preplaced PerClose Proglide (Abbott Vascular) devices or using an Angio-Seal device (St. Jude Medical) if an 8F sheath was used. The main steps of the technique are summarized in Fig 2.

**Fig 3:**
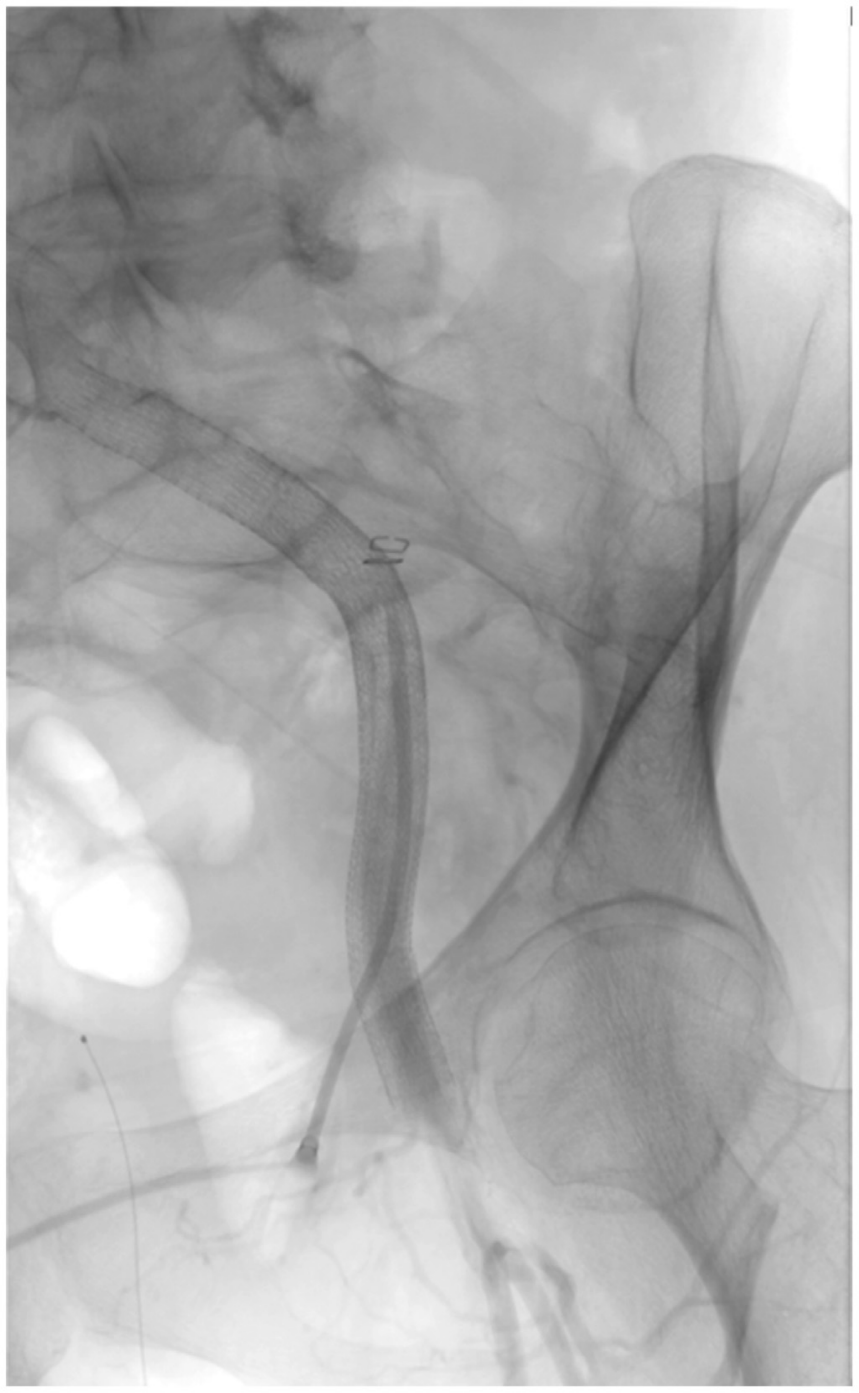
Percutaneous direct access of the endoconduit during the complex endovascular aortic aneurysm repair (EVAR) or thoracic endovascular aortic aneurysm repair (TEVAR).

**Fig 4:**
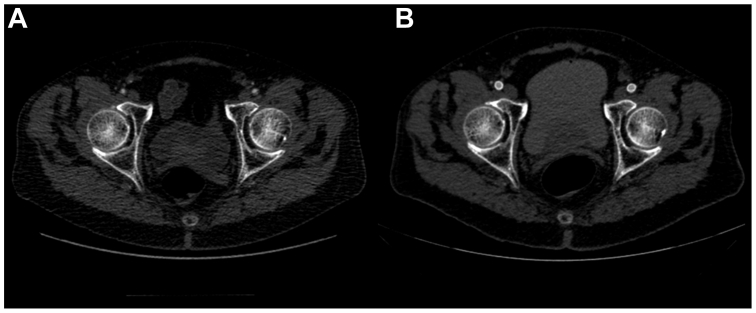
**A,** Computed tomography angiography (CTA) reveals a hostile anatomy for complex endovascular aortic aneurysm repair (EVAR) or thoracic endovascular aortic aneurysm repair (TEVAR. **B,** CTA reveals endoconduit (EC) placement to improve the future access site during complex EVAR or TEVAR.

All second-stage procedures are performed percutaneously by directly accessing the EC in the CFA under ultrasound visualization, followed by the placement of the PerClose devices (Fig 3). The EC is sequentially dilated to avoid EC disruption until the larger sheath or device is placed. The sequential gentle use of progressive larger hydrophilic dilators is key to avoiding disruption of the EC. The endovascular repair is performed as previously described.^13^ The staged complex EVAR or TEVAR procedures are usually deferred for ≥7 to 10 days after the EC procedure to allow for healing of the “paved and cracked” iliac and femoral vessels stented with the EC (Fig 4). Most patients undergo CTA a few days after the procedure to assess the results and suitability for the complex EVAR or TEVAR procedure. After the aortic procedure, follow-up with imaging (usually CTA) is obtained at 1 month, 6 months, and 1 year and annually thereafter for 5 years, including the pelvic and proximal thighs to assess the status of the EC.

## Results

During a 2-year period, 17 patients underwent staged EC before complex EVAR or TEVAR using the “up and over” technique. The median age was 79 years (interquartile range [IQR], 72-81 years). The most frequent comorbidities were smoking (100%), hypertension (85.7%), and hyperlipidemia (67.9%). The median aneurysm size was 59 mm (IQR, 54-77 mm). Aneurysms were classified as suprarenal (35%), type I to III thoracoabdominal aortic (35%), and type IV thoracoabdominal aortic (30%) aneurysms. The median diameter of the CIA, EIA, and CFA were 7 mm (IQR, 5-9 mm), 5 mm (IQR, 4-6 mm), and 6 mm (IQR, 5-8 mm), respectively. The CIA and EIA were both stented in 10 patients (58%). The IIA was excluded in three patients (17%), none of whom experienced buttock claudication after the procedure. Technical success was 94%. None of the Viabahn stent grafts deployed into the CFA kinked, occluded, or collapsed and/or resulted in limb ischemia. One EC disruption occurred with occlusion after closure and required restenting. All procedures were completed at a median follow-up of 4 weeks (IQR, 3-6 weeks) after the EC. No intraoperative vascular injuries or limb ischemia occurred.

## Discussion

This study reveals that EC before complex EVAR or TEVAR can be performed in a safe manner improve access. Our results demonstrate that the EC can be directly accessed without associated intravascular injuries. During complex EVAR, ECs are sequentially dilated to decrease the risk of vascular complications and EC disruption. In most cases, a 10-mm Viabahn stent graft for high-profile devices was used; however, with the introduction of low-profile devices, an 8-mm Viabahn stent graft could be used instead. Even with the introduction of low-profile devices, EC creation could still be useful to prevent vascular injury during complex EVAR or TEVAR.

CFA disease has traditionally been treated with open surgical approaches^14^; however, the morbidity of an incision and the time required to heal can delay the endovascular repair, with potential complications. Previous studies have reported longer operative times and more complications following open conduits compared with ECs.^15^^,^^16^ The retroperitoneal open iliac conduit approach has been related to a 2.6-fold greater blood loss, 82% longer procedure times, 1.5-day longer hospital stays, and a 1.8-fold higher rate of perioperative complications compared with the femoral approach.^17^ Open conduits during EVAR have been related to a higher mortality rate, an increase in complications, and longer postoperative stays.^18^ Furthermore, a study comparing EC and retroperitoneal open iliac conduit revealed that EC was related to lower rates of early mortality and late complications.^19^ Similarly, there were no vascular injuries or limb ischemia at short-term follow-up in our study, suggesting that the endovascular approach to treat CFA disease is feasible. Despite the advantages of endovascular conduits compared with an open approach, the associated costs are not negligible. However, the comorbidities and complications related to open techniques could increase the cost and decrease the effectiveness compared with endovascular approaches. Further studies are necessary to clarify the cost-effectiveness of this technique.

All ECs in our cohort were performed in a staged fashion, which might have contributed to the improved outcomes observed. Staged endovascular procedures have shorter postoperative recovery periods compared with open procedures.^20^ Unplanned conduits have been associated with higher blood loss, intraoperative transfusions, and overall complication rates.^21^ In our initial experience with ECs, access injuries and iliac rupture and disruptions were frequent when TEVAR or complex EVAR was performed during the same setting as the EC. The “paving and cracking” of the access vessels during EC creation could render the vessel fragile and prone to disruption during the introduction of larger sheaths. Allowing time for “healing” of the access vessels after EC creation is, therefore, prudent. Furthermore, previous studies have reported an average time between an open conduit to TEVAR or complex EVAR of 80 to 106 days.^16^^,^^21^ Our results suggest a shorter interval between EC and complex EVAR is safe, allowing for prompt repairs. However, this study does not include an open conduit group for comparison.

Hostile iliac anatomy in patients undergoing complex EVAR has been related to technically demanding procedures and increased late mortality.^8^ Severe iliac tortuosity, stenotic iliac arteries, and calcified vessels have been identified as the main reasons for ineligibility to EVAR in 28% to 47% of the patients.^22^^,^^23^ The introduction of endovascular techniques in patients with adverse iliac anatomy has been associated with improved outcomes.^19^^,^^24^^,^^25^ Nonetheless, it has been limited to retrograde approaches with restrictions on stenting distal CFA lesions. CFA stenting has been met with caution given the concerns of limiting future access options, compromising the profunda femoral artery, and risking possible stent fracture.^26^ However, previous studies have reported a 1-year primary patency of CFA stenting ranging from 80% to 92.5%^27^, ^28^, ^29^, ^30^ and 2-year primary patency from 83% to 92%.^28^^,^^31^ Bonvini et al^24^ found that angioplasty and stenting of the CFA was associated with a high success rate, a low incidence of complications, and acceptable patency. In our cohort, technical success was achieved in 94% of the patients, which is consistent with the previously reported rate of 96%.^31^ Our study revealed no 30-day mortality or intraoperative vascular injuries, in contrast to previous studies with a reported perioperative complication rate of 5% to 7.5%.^28^^,^^31^ Notably, no buttock claudication developed in our series. This might be because of the disease or highly stenotic hypogastric arteries before EC placement. Consequently, these patients likely have robust collateral circulation to perfuse the buttock, and covering the IIA did not lead to claudication symptoms. Although potential crushing, fractures, and thrombosis of stents across the groin area can be seen with bare stents, no disruption of the Viabahn stent graft in the distal EIA and CFA occurred. In this regard, the Viabahn stent graft appears to be resilient to flexion and extension across the groin area, which might be similar to what occurs in the popliteal region, where the Viabahn stent graft is frequently used.

One of the limitations of EC creation is the frequent need to cover collateral vessels in the groin area. Attempting to preserve collateral vessels by keeping the stented area proximal to the inguinal ligament could lead to accessing a diseased or stenotic CFA, which could result in disruption of the access site and collateral vessels. Therefore, and despite the sacrifice of the collaterals, stenting into the CFA is required for most patients. Although the collateral circulation might be necessary to maintain distal perfusion before treatment, it might not be as important once the iliac disease has been addressed with the EC, particularly because of the improved patency of the stent graft. The “up and over” technique allows for treatment of distal CFA lesions to facilitate more straightforward procedures and might be associated with a decrease in late mortality. However, the small sample size and short follow-up limited our evaluation of the true role of CFA stenting before complex EVAR or TEVAR.

## Conclusions

The staged “up-and-over” EC technique might improve the technical success in patients with adverse iliofemoral anatomy before complex EVAR or TEVAR. Avoiding accessing the ipsilateral CFA during the first stage improves access, avoids vascular injury, and allows for treatment of CFA disease. Long-term follow-up is required to identify possible shortcomings, contraindications, and complications of this technique.

## Disclosures

C.H.T. has been a consultant for, and received research support from, Cook Medical Inc, W.L. Gore & Associates, and Philips Healthcare. A.V.F., M.T.T., J.E.C.-F., M.S.G., and M.S.B. have no conflicts of interest.
